# Handler familiarity helps to improve working performance during novel situations in semi-captive Asian elephants

**DOI:** 10.1038/s41598-021-95048-w

**Published:** 2021-07-29

**Authors:** Océane Liehrmann, Jennie A. H. Crawley, Martin W. Seltmann, Sherine Feillet, U. Kyaw Nyein, Htoo Htoo Aung, Win Htut, Mirkka Lahdenperä, Léa Lansade, Virpi Lummaa

**Affiliations:** 1 Department of Biology, University of Turku, 20500 Turku, Finland; 2 Université Sorbonne Paris Nord, 93430 Villetaneuse, France; 3 Myanma Timber Enterprise, Gyogone Forest Compound, Bayint Naung Road, Insein Township, Yangon, 11011 Myanmar; 4 Department of Public Health, University of Turku and Turku University Hospital, 20520 Turku, Finland; 5 CNRS, IFCE, INRAE, Université de Tours, PRC, 37380 Nouzilly, France

**Keywords:** Neuroscience, Zoology

## Abstract

Working animals spend hours each day in close contact with humans and require training to understand commands and fulfil specific tasks. However, factors driving cooperation between humans and animals are still unclear, and novel situations may present challenges that have been little-studied to-date. We investigated factors driving cooperation between humans and animals in a working context through behavioural experiments with 52 working semi-captive Asian elephants. Human-managed Asian elephants constitute approximately a third of the remaining Asian elephants in the world, the majority of which live in their range countries working alongside traditional handlers. We investigated how the familiarity and experience of the handler as well as the elephant’s age and sex affected their responses when asked to perform a basic task and to cross a novel surface. The results highlighted that when novelty is involved in a working context, an elephant’s relationship length with their handler can affect their cooperation: elephants who had worked with their handler for over a year were more willing to cross the novel surface than those who had a shorter relationship with their handler. Older animals also tended to refuse to walk on the novel surface more but the sex did not affect their responses. Our study contributes much needed knowledge on human-working animal relationships which should be considered when adjusting training methods and working habits.

## Introduction

Working animals such as equines, military/sheep dogs or logging and tourism elephants, spend hours each day in close contact with humans, but factors driving cooperative interactions are still unclear. Working animals require specialised handling expertise, as they are trained to understand commands and fulfil specific tasks^1^. Training can last from several months to years depending on the species, the training methods and the tasks required. The capacity of animals and humans to understand each other is a crucial part of their working relationship^2^, and working animals require strong cognitive skills surrounding learning and memory^3^ to be properly trained.

The ability to work in different contexts is essential for the safety of working dyads and may require a strong human-animal relationship. First, working animals usually have daily routines. However, handlers must anticipate animal reactions to unusual circumstances (e.g. to novelty/fear), by gaining an understanding of their behaviour, and developing strong bonds. For instance, dog-sleigh drivers state that trust is essential in their relationships with their dogs^4^. Mahouts (elephant handlers) suggest that a three year relationship is necessary to understand an elephant’s behaviour, and eight years to develop^5^. Second, animals must be able to discriminate between different humans to maintain strong specific relationships^6,7^. Black rhinoceros *(Diceros bicornis)* and zebras *(Equus burchellii)* from zoos showed differences in their latency to respond to commands depending on the keeper involved in the task, indicating that unique dyads between zoo keepers and animals were important for behaviour^8^. Similarly, positive interactions between animals and specific handlers in zoos have previously been found to reduce abnormal and increase normal behaviours in primates^9^, and both improve reproductive success^10^ and reduce physiological stress indicators (corticoids) in felines^11^. Therefore, it is likely that handler familiarity also affects working animals’ success at performing desired tasks.

**Figure 1:**
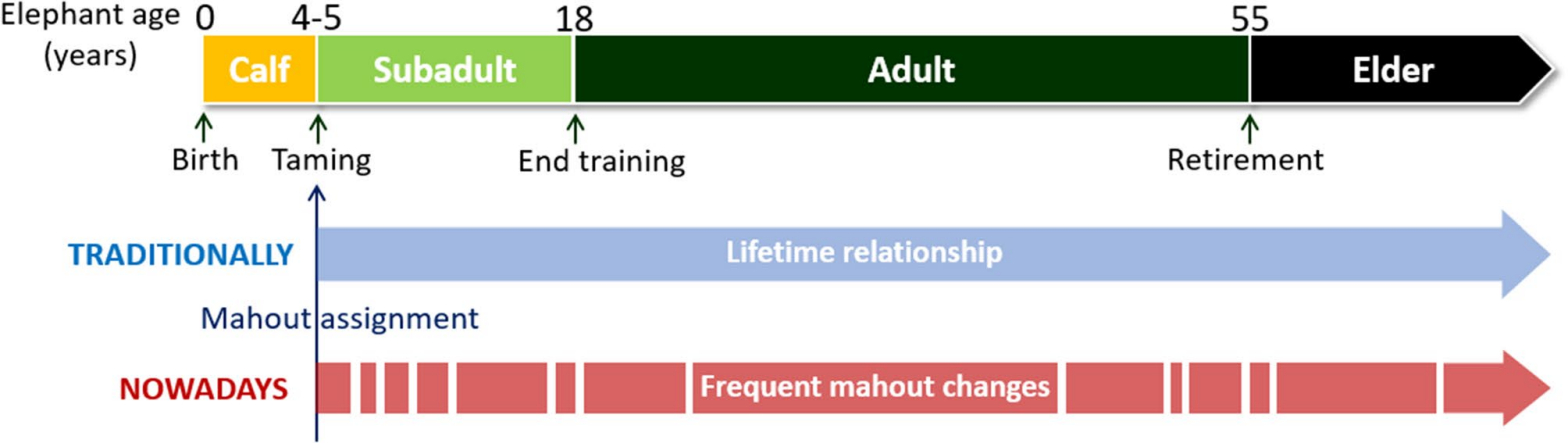
Representation of the Myanma Timber Enterprise elephant lifetime and the issues brought by the changing situation in Myanmar leading to numerous mahouts quitting over the last 10 years^17^.

We studied how handlers’ experience and familiarity with working animals affected the performance of working Asian elephants (*Elephas maximus*) in novel situations. Elephants have been used by humans for > 4000 years for religious processions, work and recently tourism^12^. They have high cognitive skills, such as self-awareness^13^, relative quantity judgment^14,15^, and the ability to discriminate between familiar and unfamiliar humans, using visual and olfactory cues^16^. The largest captive population consists of ~ 5000 semi-captive elephants in Myanmar, around 3000 of which are employed by the Myanma Timber Enterprise (MTE). Most of these elephants today are captive-born. During taming at the age of around four years, each elephant is assigned a mahout. The assigned mahout is responsible for the elephant’s training and their day-to-day care; he collects the elephant daily from the forest, ensures that it is well fed and checks for injuries or behavioural abnormalities. The elephant and his mahout develop a working relationship that traditionally lasted for a lifetime. However, the mahout profession and culture are nowadays threatened with many mahouts leaving for more profitable and less demanding city industries. Therefore, elephants today often face frequent changes of mahout, and mahouts are younger and lack experience^17^ (Fig. 1). It has been shown that positive keeper-elephant relationships in zoos can benefit the welfare of both keepers and animals^18^. Therefore, deteriorating mahout-elephant relationships could lead to decreased elephant welfare and mahout safety. Consequently, it is important to understand the mechanisms governing the mahout-elephant relationship and to investigate how the frequent mahout changes affect the quality of their interactions with the elephants. Moreover, the context of working tasks may also affect how elephants perform, as novelty is known to induce stress and is commonly used as a fearful stimulus in studies involving species with neophobic behaviours^19–21^.

We tested if the familiarity and experience of mahouts and their elephants’ sex and age (a proxy for the elephant’s own working experience as all captive-born elephants are trained from the same age) affect elephants’ responses to commands in their usual working context and in a novel context. We asked mahouts to command their elephants to cross an area marked only with wooden planks around the perimeter (usual context/control) and to cross a large plastic surface they had never walked on before (Novel context). We hypothesised that: (1) the familiarity between an elephant and the commanding mahout may affect the elephant’s success at walking on the novel surface. Pairs who have known each other for longer may understand and trust each other more and succeed more at the given task, than pairs who have never worked together before. (2) We expected elephants commanded by more experienced mahouts to have a better understanding of their commands and agree to walk on the novel surface more, whereas less experienced mahouts might be less assertive and their elephants less successful in both tasks. Finally, (3) elephants’ success at responding to their mahouts’ calls in a familiar context should increase with their age until they are fully trained^22^. On the contrary, in many species, older individuals present more neophobic behaviours than younger individuals that are more prone to exploration^23^. Hence, we (4) expect the success rate of elephants crossing a novel surface to decrease with age.

## Method

### Study population and subjects

Our study consists of 52 semi-captive Asian elephants (26 females and 26 males) owned by the Myanma Timber Enterprise. The MTE is a governmental institution, and the captive status of these elephants is approved by the Myanmar Ministry of Natural Resources and Environmental Conservation. The MTE monitors the safety and welfare of the elephants via monthly health inspections by trained veterinarians. Our study participants ranged in age from 5 to 58 years (median = 15.0; SD = 16.8), 31 of which were still in training (5 to 17 years) and 21 had ended their training (16 working and 5 retired elephants). These elephants work during state-set working hours (maximum 5–8 h a day) under the guidance of an assigned mahout who is responsible for their everyday care. The elephants are kept in groups of approximately six elephants and an experienced head mahout manages the operations of the group. At night, individuals forage unsupervised in nearby forests where they can interact and mate either with each other or with wild elephants. There is no control of their reproduction, hence the ‘semi-captive’ definition of these animals. During their non-working time they can develop and express their natural behaviours without the stress of a captive and confined environment, known to be detrimental to elephant welfare^24,25^. Their diet is minimally supplemented (occasional fruit, salt and rice when travelling). The MTE maintains detailed logbooks on every individual elephant with reliable records of: birth date, offspring information, sex, origin (captive born/ wild caught) as well as health reports from bi-monthly vet inspections of working ability and medical condition. Calves are separated from their mother and tamed at 4–5 years of age. The initial taming takes four weeks and each calf is allocated an individual mahout^26,27^. They are trained and used for light work duties until they start working full-time at the age of 18 years, and they are retired at the age of 55.

### Mahouts information

We interviewed mahouts about their work-related information such as their overall experience working as a mahout and the time since they were assigned to their elephant at the time of the experiment (referred to as “relationship length”)^17^. The overall working experience of the mahouts varied from 1 month to 14.5 years, and since there was an uneven distribution of experience lengths, this variable was divided into three groups: less experienced (1 month–1 year; n = 10), experienced (1–4 years; n = 18) and more experienced (> 4 years; n = 23) mahouts. On the day of the tests, the assigned mahouts of ten elephants were unavailable to participate in the tests. Therefore, these elephants were tested with a mahout unknown to them with whom they had never worked before (each had a different unknown mahout paired with them). The relationship length between the focal elephant and the mahout calling was therefore categorised into three groups: unknown mahout (relationship length = 0; n = 10), known for less than a year (1 week–1 year; n = 23) and known for more than a year (1–11 years; n = 19).

### Experimental design

The behavioural tests were performed between the 28th of March and the 7th of April 2018 at five different MTE elephant camps. As these dates correspond to the hot season in Myanmar, the tests were only performed during the morning to avoid the elephants being exposed to too high temperatures. Mahout-elephant dyads participating in the experiments were not specifically chosen. Participating mahouts were those present in the camps on the days of the experiments. Two tests were performed (control and novel surface), each elephant was tested once per test. 42 elephants performed in both tests with their assigned mahout and 10 elephants without their assigned mahout present were tested with a mahout unknown to them in both tests.

#### Calling test (Control)

We built an arena by placing wooden planks around the perimeter of a rectangular 8 m × 3 m area on the natural floor of each elephant camp^22^. First, the elephants were instructed by the mahout to stand and wait (held by another mahout if necessary) on one side of the arena until the mahout took position on the opposite side of the arena. Then the mahout gave vocal commands for the elephant to cross the arena lengthwise. A video camera (Sony HDR-CX405) was used to record the experiment. Videos were analysed using the free Behavioural Observation Research Interactive Software (BORIS)^28^. The elephant's success at joining the mahout by crossing the arena was recorded as a binary response (0/1).

#### Novel surface test

The goal of this experiment was to introduce the focal elephant to a novel stimulus by asking the animal to walk on a surface it had never encountered before. This experimental design has been adapted from similar experiments performed on horses^29^. We placed a 6 m × 3 m silver plastic sheet on the ground in an unobstructed open space. The elephant was positioned by the calling mahout to stand in front of the shorter end of the surface. After having instructed the elephant to hold their position, the mahout walked across the silver plastic sheet lengthwise (walking beside the novel surface could induce the elephant to do the same). Once on the other side of the sheet, the mahout vocally commanded the elephant to come to him. We recorded the elephant's success at joining the mahout by walking on the surface (four feet touching the surface) as a binary response (0/1, again analysing videos using BORIS). Four of the elephants did not cross the surface lengthwise and just walked on a part of it to join their mahout. This number was considered too small to be a separate category in statistical analyses and as they walked with their four feet on the surface, we judged them as having successfully finished the task. Four other elephants put one foot on the surface and then decided not to walk on it; these we considered to have failed the task. We excluded 10 elephants from the novel surface analyses who had not responded to the call of their mahout in the control task. Since these 10 individuals did not respond to a simple call, analysing their response to the novel surface would be difficult. We would not be able to distinguish, for example, if a lack of reaction was due to misunderstanding the call or their reluctance towards the novel surface.

### Statistical analyses

Data are given in Online Resource 1. All analyses were performed with the statistical software R, version 3.6.3^30^ and figures were created using the *ggplot2* package^31^. To investigate how task success (1: success/0: fail) was affected by elephant age and sex and mahout experience and relationship length, two Bayesian regression models were fit using the *brm* function from the *brms* package^32^ with Bernoulli distributions. We employed a Bayesian framework since our sample size was relatively small and underlying data structure (singular fit) limited restricted maximum likelihood model convergence. We used weakly informative priors set automatically by the *brm* function. Since test sessions occurred on seven different days in four different locations, we included the test date as a random factor to control for variation due to the weather or location in both models. In addition, we included individual ID nested in date of test as a random factor to account for individual variation, since the same elephants were measured in both tests. We examined effect sizes of regression coefficients, and judged their importance based on the credible intervals, where effects were considered significant if the credible intervals did not encompass zero, and a trend if the credible intervals encompassed zero but one of the intervals was between − 1 and 1.

We checked for potential collinearity with a bivariate analyses using Spearman correlation tests in between the elephant age, the elephant sex, the mahout experience and the mahout-elephant relationship length. There was no collinearity among the covariates and therefore they could be included together in the models.

To avoid over-parameterisation of the models, we assessed mahout and elephant predictors in separate models^33,34^. The first two models assessed the effect of the mahout predictors (mahout experience and relationship length) on the success of elephants in the control task (model 1; N = 51) and in the novel surface task (model 2; N = 41). The sample size and the distribution of the data did not allow us to test for a two-way interaction between the mahout experience and the relationship length, but raw data are presented in Table 1. The last two models tested whether elephant’s age and sex affected their success in the control task (model 3; N = 52) or in the novel surface task (model 4, N = 42). We tested the effects of sex and age as main predictors to examine whether elephants’ responses to the novel surface or control tasks were different for males and females or depended on their age. Model selection was performed according to leave-one-out cross-validation^35^ using the *LOO* function from the *brms* package^32^.

**Table 1:** Number of successes of elephants at the novel surface task for the different dyads for each crossed category.

Success/total N	Unknown mahout	Known for less than a year	Known for more than a year
Less experienced	0/3	2/5	0/0
Experienced	0/1	2/7	2/5
More experienced	1/3	3/8	5/9

To assess the differences between different levels of the variables, the categorical variables (mahout experience and relationship length) were re-levelled to vary the intercept in the models and to obtain the statistical values for the effect of each level.

### Ethical standard

Our study was conducted following the ethical guidelines of the University of Turku, and has been approved by the Myanmar Ministry of Natural Resources and Environmental Conservation according to an established Letter of Agreement. This study did not contain any procedure that would require a project license according to the Finnish National legislation Act 497/2013 and Decree 564/2013 on the protection of animals used for scientific or educational purposes or the EU Directive 2010/EU/63 on the protection of animals used for scientific purposes.

## Results

### Relationship between overall mahout experience and relationship length, and task success

#### Model 1 (control task)

**Figure 2:**
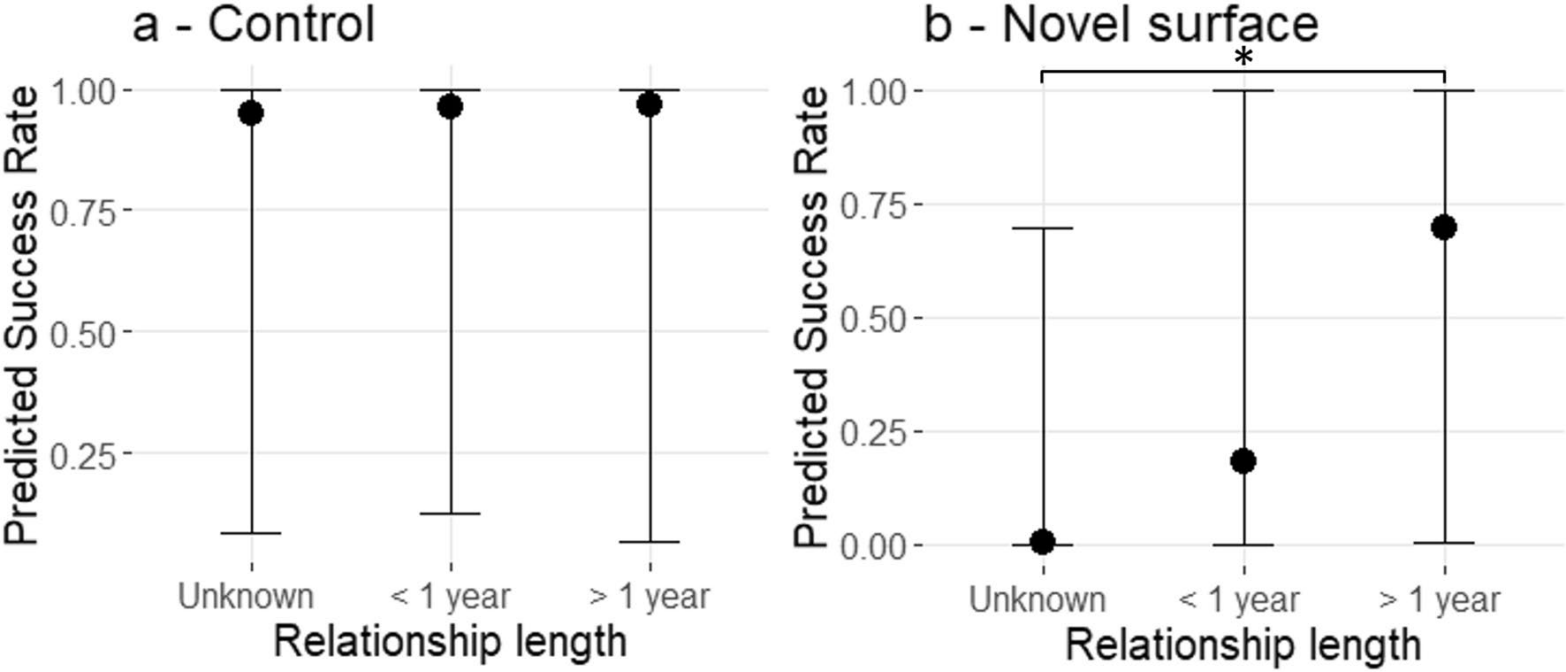
Predicted success rate depending on mahout-elephant relationship length. (**a**—control, **b**—Novel surface) Bars represent the 95% credibility intervals. (Bayesian regression models from Table 2). “*” means significant differences in between the variables from the extremity of the bracket.

**Figure 3:**
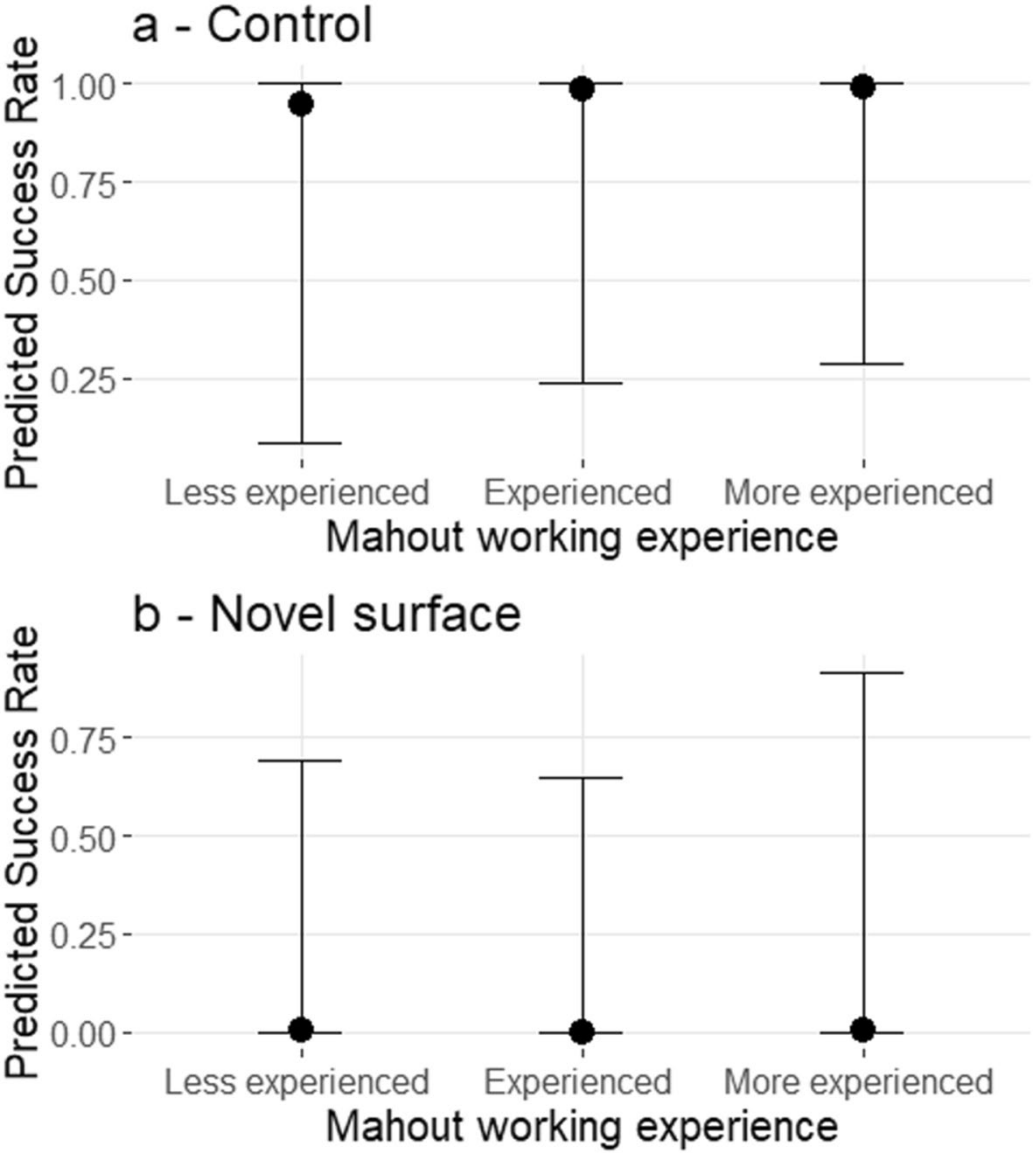
Predicted success rate depending on mahout working experience. (**a**—control, **b**—Novel surface). Bars represent the 95% credibility intervals. (Bayesian regression models from Table 2).

Overall, 82% of the elephants responded to the call of their mahout but mahouts’ parameters did not affect the response of elephants to calling in the control task. Elephants did not perform differently depending on whether they were called by unknown mahouts, mahouts known for less than a year or mahouts known for more than a year (Fig. 2a; Table 2—model 1). Similarly, their responses did not differ depending on the calling mahout’s total working experience (Fig. 3a; Table 2—model 1).

**Table 2:** Bayesian regression models analysing elephant success according to their relationship length with the calling mahout (RL: Unknown, < 1 year = Known for less than a year, > 1 year = known for more than a year) and the mahout’s working experience (EXP: Less experienced, Experienced, More experienced). Results from Model 1 are for the control task (N = 51) and results from Model 2 are for the novel surface task (N = 41) Bold = statistically significant variables (CI’s do not encompass 0), italic = tendency effects.

		Explanatory	Estimate ± SE	Lower 95% CI	Upper 95% CI
Control	Model 1 Intercept 1	*Intercept (RL: Unknown / EXP: Less experienced)*	*3.57 ± 3.80*	* − 2.36*	*11.65*
		RL: < 1 year	0.14 ± 1.67	− 3.19	3.54
		RL: > 1 year	0.26 ± 1.76	− 3.30	3.67
		EXP: Experienced	1.25 ± 1.83	− 2.09	5.23
		EXP: More experienced	1.74 ± 2.00	− 1.80	5.94
	Model 1 Intercept 2	*Intercept (RL: < 1 year / EXP: Experienced)*	*4.66 ± 3.52*	* − 0.67*	*13.57*
		RL: Unknown	− 0.07 ± 1.72	− 3.42	3.38
		RL: > 1 year	0.14 ± 1.51	− 2.76	3.08
		EXP: Less experienced	− 0.99 ± 1.78	− 4.77	2.39
		EXP: More experienced	0.58 ± 1.27	− 1.87	3.07
	Model 1 Intercept 3	***Intercept (RL: > 1 year / EXP: More experienced )***	***5.64 ± 3.68***	***0.01***	***13.90***
		RL: Unknown	− 0.34 ± 1.79	− 3.71	2.61
		RL: < 1 year	− 0.21 ± 1.47	− 3.03	3.23
		EXP: Experienced	− 0.57 ± 1.25	− 3.08	2.02
		EXP: Less experienced	− 1.63 ± 1.97	− 5.58	2.07
Novel surface	Model 2 Intercept 1	*Intercept (RL: Unknown / EXP: Less experienced)*	* − 9.06 ± 8.30*	* − 26.81*	*0.82*
		RL: < 1 year	7.58 ± 8.59	− 1.25	29.21
		**RL: > 1 year**	**10.17 ± 9.23**	**0.29**	**33.25**
		EXP: Experienced	− 0.85 ± 2.03	− 5.19	2.87
		EXP: More experienced	1.06 ± 1.56	− 1.83	4.40
	Model 2 Intercept 2	*Intercept (RL: < 1 year / EXP: Experienced)*	* − 2.59 ± 4.22*	* − 11.11*	*6.24*
		RL: Unknown	− 7.40 ± 8.29	− 29.04	1.51
		RL: > 1 year	2.54 ± 1.95	− 0.56	6.84
		EXP: Less experienced	0.89 ± 2.08	− 3.34	4.91
		EXP: More experienced	1.93 ± 1.52	− 0.68	5.27
	Model 2Intercept 3	*Intercept (RL: > 1 year / EXP: More experienced)*	*2.04 ± 3.98*	* − 4.53*	*11.26*
		**RL: Unknown**	** − 9.85 ± 8.00**	** − 30.60**	** − 0.45**
		RL: < 1 year	− 2.48 ± 2.20	− 7.28	1.05
		EXP: Experienced	− 1.87 ± 1.53	− 5.19	0.70
		EXP: Less experienced	− 0.90 ± 1.55	− 4.31	1.89

#### Model 2 (novel surface task)

First of all, 38% of the individual agreed to walk on the novel surface, in contrast to the simple control task, the elephants’ responses to the novel surface task were significantly affected by the elephant-mahout relationship length. Elephants called by mahouts known for more than a year successfully walked on the novel surface more often than elephants called by unknown mahouts (Estimate = 10.17 ± 9.23; lwr | upr 95% CI = 0.29 | 33.25). They also tended to be more successful than elephants called by mahouts known for less than a year (Estimate = 2.54 ± 1.95; lwr | upr 95% CI = − 0.56 | 6.84). There were however no significant differences in the responses of elephants called by unknown mahouts and those called by mahouts known for less than a year (Estimate = − 7.40 ± 8.29; lwr | upr 95% CI = − 29.04 | 1.51) (Fig. 2b; Table 2-model 2). On the contrary, we did not observe a significant effect of overall mahout experience on the success rate of elephants crossing the novel surface. Elephants were not more successful when called by a more experienced mahout compared to the other experience categories (Fig. 3b; Table 2—model 2).

### Relationship between elephant sex and age, and task success

#### Model 3 (control task)

**Figure 4:**
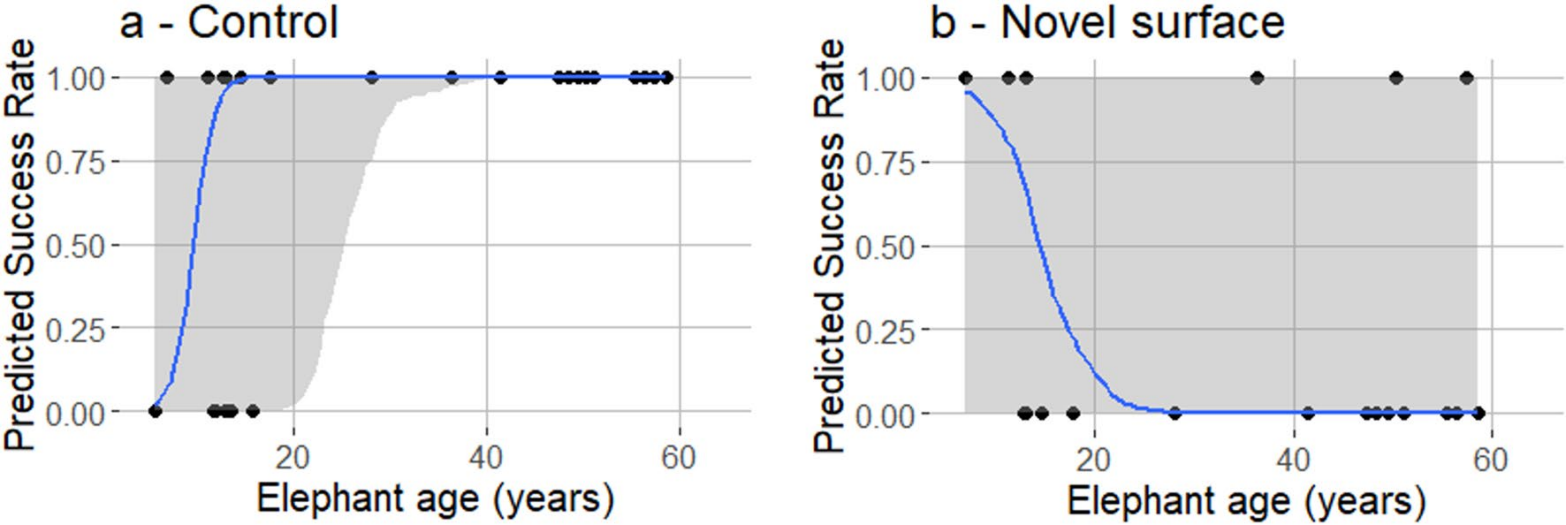
Predicted success rate depending on the task and elephant age (years), (**a**) control; (**b**) Novel Surface. Shaded areas show the 95% credibility intervals (Bayesian regression models 3 and 4 from Table 3).

During the control task older elephants were more likely to respond successfully than younger elephants (Estimate = 1.19 ± 1.03; lwr | upr 95% **CI = 0.03 | 3.62**), with the success rate being 50% for 10-year-old elephants but reaching 100% for 23-year-olds (Fig. 4a; Table 3—model 3). There was no interaction between elephant sex and the task success; both sexes responded similarly in the control task, and nor was there an overall difference in the success rate of males and females (Table 3—model 3).

**Table 3:** Bayesian regression models analysing elephant success in relation to their age and sex (M = males, F = females) depending on the task (control—Model 3, N = 52; novel surface – Model 4, N = 42). All models included elephant identity nested in the date as random factors. Priors were automatically set by the brms function. Bold = Significant effects (CI’s do not encompass 0), italic = tendency effects.

	Explanatory	Estimate ± SE	Lower 95% CI	Upper 95% CI
Control Model 3	*Intercept (sex: F)*	*− 11.62 ± 18.61*	*− 51.99*	*14.97*
	**Elephant age**	**1.19 ± 1.03**	**0.03**	**3.62**
	Sex: M	11.87 ± 13.56	− 4.62	41.81
Novel surface Model 4	*Intercept (sex: F)*	*12.19 ± 42.10*	*− 33.73*	*90.97*
	Elephant age	− 0.68 ± 1.22	− 3.57	0.41
	Sex: M	2.46 ± 28.35	− 41.12	48.07

#### Model 4 (novel surface task)

During the novel surface test, the results showed the opposite effect, with elephants tending to be less successful as they aged, though the effect was not significant but can be considered a tendency (Estimate = − 0.68 ± 1.22; lwr | upr 95% CI = − 3.57 | 0.41) (Fig. 4b; Table 3—model 4). There was no interaction between elephant sex and the task success; both sexes responded similarly in the novel surface task, and nor was there an overall difference in the success rate of males and females (Table 3—model 4).

## Discussion

This study highlights that when confronted with novelty, elephants with unknown mahouts and those with shorter relationships with their mahouts agreed to walk on the novel surface less often than elephants called by a mahout they had known for more than a year. This effect was not present in the control task, when there was no novelty involved. Elephants older than 20 years all successfully responded to basic commands from their mahout in the control task, but when a novel stimulus was introduced to the task, older elephants tended to fail more than younger elephants. The overall working experience of the calling mahout did not affect the success rate of elephants in the control task or the novel surface task. The results have relevance for improving the work safety and welfare of captive elephants in free contact systems worldwide, that constitute > 20% of remaining Asian elephants^36,37^, as well as highlighting the importance of longer relationships between working dyads in promoting trust particularly in novel situations for other working species.

The novel surface proved to be an effective stimulus to initiate variation in the elephants’ responses to simple commands. Most of the elephants (82%) responded to the call of their mahout when crossing a familiar arena but less than half of them (38%) agreed to walk on the novel surface. This result indicates that novelty may strongly affect elephants’ working efficiency and the communication between mahouts and their elephants. The capacity of animals and humans to communicate with each other is a crucial part of their working relationship^2^. In 1997 Lair^12^ estimated that there were about 10 to 20 MTE mahouts killed annually in work accidents. In such dangerous professions, the mutual understanding between the handler and the animal is vital.

We found that elephants called by unknown mahouts or mahouts known for less than a year were less successful at the novel surface task than elephants who had known their mahout for more than a year. When novelty is introduced to the task, it is likely that elephants perform better when they have known their mahout for a longer time, as they will have had more time to establish trust in their relationship and will have learnt how to adjust to each other’s demands. These results are congruent with Crawley et al.’s (2021)^22^ observations that elephants respond more successfully and faster to mahouts they have known for a longer time. An interesting complement to this study would be to assess the quality of the relationship by investigating the training methods used by the mahouts (training based on reward or punishment) or the time mahouts spend with their elephant outside of working/training time engaged in positive interactions such as feeding or bathing (traditionally mahouts bathe their elephants in the river every morning). For example, one of the reasons for differences in human-animal relationships between different farm management systems is the variation in the number, duration, and nature of daily interactions between handlers and animals^38^. In horses, training based on reward promotes positive behaviours towards the handler which extends to novel people^39^ and regular grooming sessions with owners create positive and durable relationships^40^. Quantifying the amount of time spent engaged in positive interactions and the number of positive interactions between mahouts and their elephants would be useful indicators of relationship quality.

Animal training is one of the most important factors to consider when studying working performance, as animals must learn a range of oral and gestural commands relating to a task in order to accomplish it successfully. In zoos, black rhinoceros and zebras with professional daily training respond better to their keepers’ cues than partially trained and untrained individuals^41^. This is consistent with our findings, as during the control task the success rate of elephants strongly increased as they aged until the age of 20 after which none of the older elephants failed the test. This threshold corresponds to the age at which the training period of MTE elephants (age 5–18 years) ends. This age effect disappeared in the novel surface task, and even tended to show the opposite, with older elephants being more reluctant to walk on the novel surface than younger individuals, indicating that past training cannot predict success rate when a novel stimulus is involved. The refusal of older individuals to cross the novel surface could be explained by neophobia (aversion to novelty); in the wild, many species avoid interacting with novel elements as a survival strategy^42^. Most studies have found lower neophobia and higher exploration in juveniles than adults^23,43,44^. Juveniles often express more explorative behaviours whilst learning what to avoid through time, leading to more neophobic tendencies among older individuals^45^. The earlier an object is encountered in an individual’s life, the more common it is perceived to be and the longer the individual will exploit this knowledge in the future^23^. The juvenile exploratory period should extend longer in long-lived animals, which was supported by our findings. Elephants can reach 70 years of age^46^ and although older elephants tended to be more reticent in crossing the novel surface than younger individuals, this was not statistically significant. The number of retired elephants (55 years and older) in our sample was too small to include them as a separate age category in analyses but four out of the five retired elephants failed to cross the novel surface. Future studies would benefit from increasing the number of older animals for further exploration of age effects in the novel surface task.

Intriguingly, there was no effect of overall mahout experience on elephant responses. It seems that mahout experience has less impact on working efficiency than their specific relationship lengths with elephants, which corroborates findings by Srinivasaiah (2014)^5^: unfamiliar mahouts who knew all the commands could not be assured of an elephant's compliance to a command. Unfortunately, we were unable to assess the effect of the interaction between mahout experience and relationship length on elephants’ success at the tests, but the raw data indicate that elephants never walked on the novel surface for unknown and less experienced mahouts whilst almost 50% of elephants walked on the surface for very experienced mahouts who had known their elephant for more than a year. The combination of a strong working experience and a long relationship with the elephant may lead to better communication and understanding between the elephant and their mahout. Crawley et al.^17^ observed that current mahouts in the MTE tend to lack experience and often resign for other jobs quite easily, which may reflect their reluctance to invest time in an undesirable job. Because of the lack of trained mahouts, most new mahouts start working with their elephant before having a proper training period. This is concerning as it can directly affect mahout and elephant safety. According to several studies, a handler’s knowledge, their experience of particular animals and their job satisfaction can strongly impact handlers’ behaviour towards animals, suggesting that these factors could therefore influence the human-animal relationship and in turn animal care^8,47,48^.

## Conclusion

Novelty in a working context, where animals are used to a certain routine, may affect working efficiency, even for trained and experienced animals. We observed that older individuals tended to be more reluctant to cross a novel surface. This finding corroborates several other studies suggesting a decrease in curiosity with age. The familiarity of the handler affected cooperation in the dyad: elephants were more reluctant to cross the novel surface when they had a shorter relationship with their assigned mahout, compared to elephants commanded by mahouts known for more than a year. Maintaining longer relationships between working dyads of mahout and elephants could promote trust and improve understanding between humans and elephants. To avoid abrupt changes of mahouts, elephants could be paired with more than one mahout, so that they would develop strong relationships with more than one mahout. If an assigned mahout is not available to work, other assigned mahouts could safely work with the specific elephant. This could also promote a slower introduction of new mahouts to the elephant and a transfer of knowledge between the elephant keepers. As suggested in Hosey and Melfi’s review (2014)^49^ further investigation of the human-animal relationship is needed to understand whether it has positive, neutral or negative consequences, both for humans and for animals. Recently, Ward and Hosey (2020)^50^ highlighted the need to combine animal welfare research in agricultural, laboratory and zoo systems that all maintain captive animals. This should also be extend to draft animals for which the human-working animal relationships is an important part of their everyday life with intensive interactions. More such studies are needed to provide a better understanding of the mechanisms underlying human-animal comprehension. This information should be integrated into management to adjust animal training methods and working habits, reduce stress and conflicts and ultimately improve elephant welfare and working performance, as well as the security of both handlers and animals.

## Supplementary Information

Supplementary Information.

## Data Availability

All data generated or analysed during this study are included in the supplementary material.
